# Friend retrovirus infection induces the development of memory-like natural killer cells

**DOI:** 10.1186/s12977-018-0450-1

**Published:** 2018-10-06

**Authors:** Elisabeth Littwitz-Salomon, Thanh Nguyen, Simone Schimmer, Ulf Dittmer

**Affiliations:** 1 University of Duisburg-Essen, Essen, Germany; 2 Institute for Virology, University Hospital Essen, University of Duisburg-Essen, Essen, Germany

**Keywords:** Natural killer cells, Immunological memory, Friend virus, Retrovirus, Memory NK cells, Antigen specificity

## Abstract

Traditionally, NK cells belong to the innate immune system and eliminate virus-infected cells through their germline-encoded receptors. However, NK cells were recently reported to possess memory-like functions that were predominantly provided by hepatic NK cells. Memory properties were mainly documented in contact hypersensitivity models or during cytomegalovirus infections. However, the precise role and the physiologic importance of memory-like NK cells during retroviral infections are still under investigation. Here, we show that Friend retrovirus (FV) infection of mice induced a population of phenotypically memory-like NK cells at 28 days post infection. Upon secondary antigen encounter, these NK cells showed an increased production of the pro-inflammatory cytokines IFNγ and TNFα as well as the death ligand FasL in comparison to naïve NK cells. Furthermore, we found an augmented elimination of antigen-matched but not antigen-mismatched target cells by these memory-like NK cells. In adoptive cell transfer experiments, equal antiviral activities of splenic and hepatic memory-like NK cells during the late phase of acute FV infection were found. Our results strongly imply the existence and antiviral activity of spleen and liver memory-like NK cells in FV infection, which efficiently respond upon secondary exposure to retroviral antigens.

**Electronic supplementary material:**

The online version of this article (10.1186/s12977-018-0450-1) contains supplementary material, which is available to authorized users.

## Main text

Natural killer (NK) cells classically belong to the innate immune system and fight cancers as well as intracellular pathogens. They sense altered cells with their germline-encoded cell surface receptors mainly through changes in the major histocompatibility complex (MHC) class I expression or via the upregulation of ligands for their activating receptors. Activated NK cells eliminate target cells by the release of cytotoxic granules that contain granzymes and perforin. Granules are exocytosed into the immunologic synapse, ultimately inducing apoptosis [1]. NK cells also express death receptor ligands on the cell surface, e.g. Tumor necrosis factor (TNF)-related apoptosis-inducing ligand (TRAIL) and FasL, that result in elimination of abnormal cells upon binding with its death receptors [2]. In addition, NK cells produce a broad range of pro-inflammatory cytokines such as interferon (IFN) γ, Tumor necrosis factor (TNF) α and Granulocyte–macrophage colony-stimulating factor (GM-CSF), which can interfere with tumor cell proliferation or virus replication [3].

Although NK cells have traditionally been considered as innate lymphocytes with no memory formation, O´Leary and colleagues described for the first time memory-like features of NK cells in a contact hypersensitivity (CHS) model [4]. Immunological memory is classically defined as the ability to rapidly respond and provide protection against previously encountered pathogens. The process of memory formation includes the longevity of memory cells, which is usually initiated during the acute infection, and recall responses upon a secondary challenge with the identical pathogen. Classical immunological memory that is usually attributed to T and B cells is divided into three phases: (1) expansion of immune cells; (2) contraction phase, in which the majority of effector cells undergo apoptosis; (3) memory phase with long-living, self-renewing subsets of immune cells [5]. Similar to NK cell memory to haptens, skin sensitization with virus-like particles containing antigens from HIV-1, influenza or vesicular stomatitis virus resulted in memory NK cell responses, although NK cells lack antigen-specific rearranged receptors [6]. Hence, for the memory formation of these distinct immune cells antigen-specificity is required that should differ between NK cells and T cells. Currently, two concepts of antigen specific recognition of virus-infected target cells by NK cells are being postulated. (1) For the expansion and differentiation of adaptive NK cells, viral proteins can be recognized by their activating receptors, e.g. the MCMV-encoded protein m157 that directly interacts with the activating NK cell receptor Ly49H in the mouse [7]. In human cytomegalovirus (CMV) infection, the activating receptor CD94-NKG2C^+^ recognize the non-classical MHC I molecule HLA-E as cognate ligand [8]. HLA-E surface molecules require peptide loading for stabilization, which are provided by the host or by HCMV-encoded peptides (e.g. UL40) that mimic peptide sequences of host [9, 10]. Hammer and colleagues demonstrated that a single substitution in a UL40-derived peptide resulted in altered NK cell binding to HLA-E and distinct UL-40 peptides mediated different degrees of NK cell proliferation [11]. (2) The second concept is based on the role of inhibitory receptors on NK cells, which usually recognize MHC I molecules [12]. However, on the cell surface MHC I molecules are always associated with a bound peptide within the binding groove. Virus-induced changes in the peptide reservoir presented by MHC I molecules can alter the strength of NK cell receptor binding to the inhibitory MHC I, hence, influencing NK cell inhibition. This can ultimately lead to NK cell activation and target cell lysis if a viral peptide in the MHC I binding groove strongly interferes with MHC I recognition [13]. Interestingly, single amino acid changes in the presented peptide can result in abolished or enhanced MHC I binding and subsequently triggers or dampens the NK cell degranulation, e.g. in HIV-1 infection [14, 15].

Infections with cytomegaloviruses (CMV) or simian immunodeficiency virus (SIV) and also haptens were shown to induce an antigen-specific NK cell memory [7, 16]. Effective memory NK cells can be found in spleens and livers of SIV- and MCMV-infected animals, although hapten-induced memory NK cells as well as memory NK cells from influenza-infected mice were only present in livers [4, 7, 16, 17]. Especially hepatic NK cells have been shown to possess characteristics of adaptive immune cells such as clonal expansion, longevity and robust recall responses including the production of IFNγ and degranulation [7]. For the induction of adaptive NK cells also pro-inflammatory signals are essential. Especially IL-12 but also IFNα are important for the induction of memory NK cells [18, 19]. The cytokines IL-12, IL-15 and IL-18 induce long-lived NK cells, which produce IFNγ after restimulation, whereas they do not show increased cytotoxicity [20].

While there are several studies on memory-like NK cells in CHS and CMV models, not much is known about adaptive NK cells in retroviral infections. To address this issue, we explored the functionality and phenotypical characteristics of memory-like NK cells in FV-infected mice. Here we demonstrate the existence of memory-like NK cells during FV infection. We revealed the occurrence of adaptive NK cells in FV-infected bone marrow, spleens and lymph nodes as well as in the liver. Their killing of target cells was antigen-specific and correlated with the production of pro-inflammatory cytokines as well as the expression of death receptor ligands.

### Phenotyping of memory-like NK cells from FV-infected mice

**Fig. 1:**
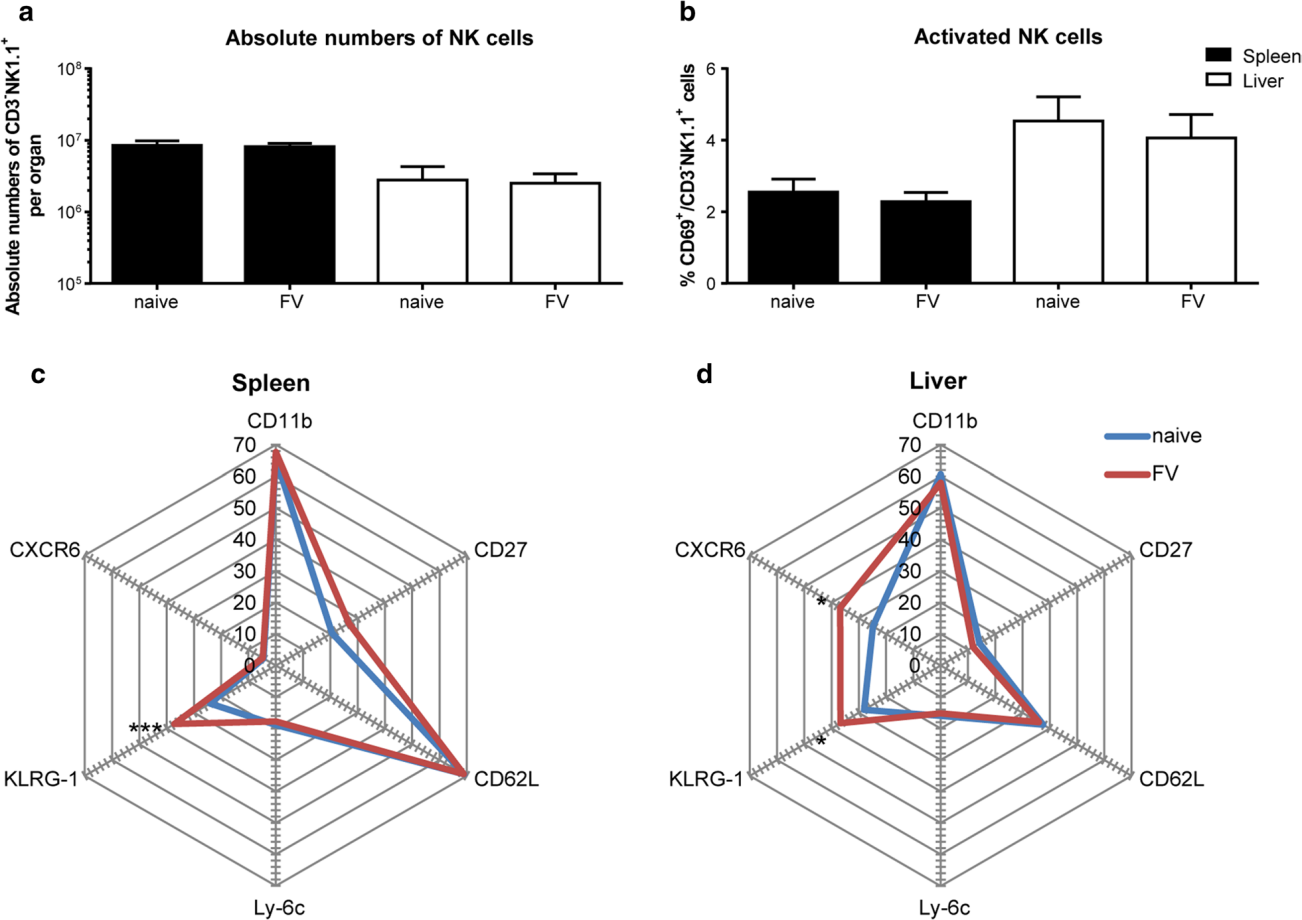
Characterization of adaptive NK cells in the late phase of acute FV infection. C57BL/6 mice were infected with FV and sacrificed at 28 dpi. Naïve animals were used as control. Single cell suspensions were prepared from spleens and livers and cells were counted. NK cells were identified by gating on lymphocytes, singlets, viable cells, CD3^−^ cells and NK1.1^+^ cells. Absolute numbers of spleen (black) and liver (white) NK cells were displayed in (**a**) (± SEM). Activation of splenic (black) and hepatic (white) NK cells was measured with the early activation marker CD69 (**b**). The mean percentage of NK cells that express memory-associated markers such as CD11b, CD27, CD62L, Ly-6C, KLRG-1 and CXCR6 are shown in (**c**) for splenic NK cells and in (**d**) for hepatic NK cells. At least eight animals from at least 3 independent experiments were used for the analysis. Statistically significant differences between naïve and FV-infected group were analyzed by an unpaired t test and are indicated by *p < 0.05 and ***p < 0.001

NK cells are traditionally classified as innate immune cells but since 2006 they are also categorized as cells with memory functions [4]. Various markers are described for the characterization of memory-like NK cells. Memory-like NK cells upregulate the expression of Ly-6c and CD62L, whereas effector NK cells express only intermediate levels of Ly-6c and CD62L [21]. The molecule KLRG-1 can be used to determine memory T cells as well as memory-like NK cells [21]. CD11b and CD27, usually used to characterize developmental stages of NK cells [22], give hints towards memory-like NK cell activity [4, 23] and the chemokine receptor CXCR6 was demonstrated to be important for the antigen-specific memory of hepatic NK cells [6]. Interestingly, memory functions were often exclusively executed by hepatic NK cells [4, 6, 17]. To address the question whether we can find a specific NK cell phenotype in mice infected with a retrovirus, we first analyzed the absolute numbers (Fig. 1a) and the activation (Fig. 1b) of all NK cells at 28 dpi directly ex vivo. As we were also interested in organ-specific differences, we analyzed spleen and liver NK cells. We detected higher numbers of NK cells in the spleen than in the liver, but these numbers were not affected by FV infection (Fig. 1a). The analysis of the early activation marker CD69 revealed unaltered levels of NK cell activation after FV infection in both organs (Fig. 1b). To address the question whether the markers described above could be used to identify and characterize memory-like NK cells during late phase of acute FV infection, we analyzed the percentage of NK cells positive for these markers in the spleen (Fig. 1c, Additional file 1: Fig. S1) and liver (Fig. 1d, Additional file 1: Fig. S1). Although we did not see any significant differences for CD11b, CD27, CD62L and Ly-6c expression in splenic or hepatic NK cells from naïve or FV-infected mice, we detected an increased percentage of KLRG-1^+^ NK cells in FV-infected mice in both organs. The percentage of CXCR6^+^ NK cells was significantly higher in hepatic NK cells after FV infection.

Taken together, only the enhanced expression of KLRG-1 and CXCR6 on NK cells after FV infection suggested the induction of a memory-like NK cell population.

### NK cells in FV-infected animals are highly cytotoxic after re-challenge with FV-induced tumor cells

**Fig. 2:**
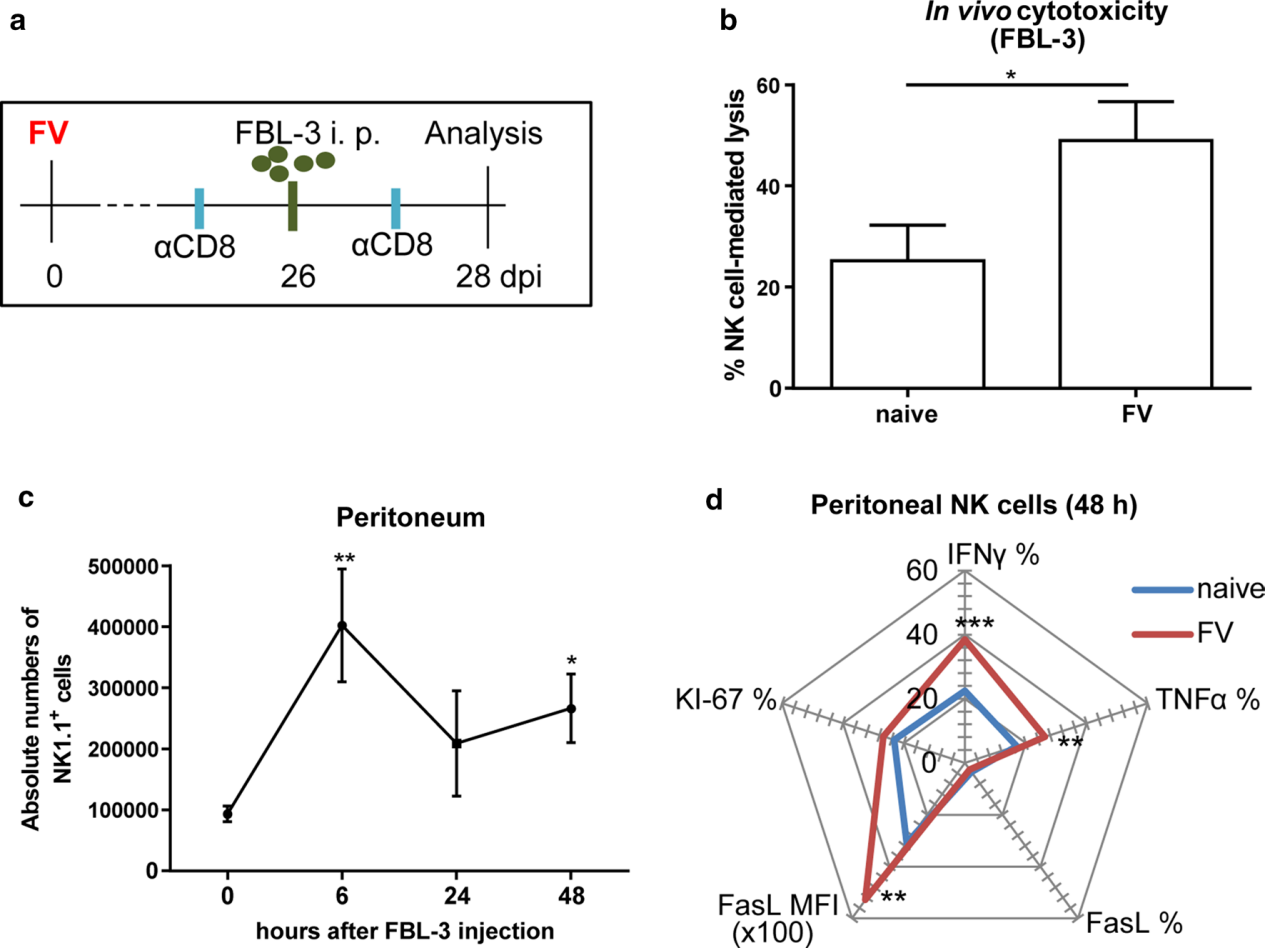
Cytotoxic functions of adaptive NK cells after challenge with FV-induced target cells. C57BL/6 mice were infected with FV and treated as indicated in box (**a**). Naïve mice were also depleted for CD8^+^ T cells and injected i. p. with CFSE^+^ FBL-3 cells. After 48 h incubation, peritoneal lavage was performed and cells were counted. CFSE^+^ FBL-3 cells were measured at flow cytometer. The percentage of NK cell-mediated lysis of FBL-3 cells was calculated and is displayed in (**b**) (± SEM). FBL-3 cell killing in NK cell-depleted mice was used for calculations. Statistically significant differences were analyzed by an unpaired *t* test. At least eleven animals from three independent experiments were used for the analysis. Peritoneal lavage was performed after 0, 6, 24 and 48 h after FBL-3 cell injection into naïve and FV-infected mice (pooled data). NK cell numbers were determined and are shown in (**c**) (± SEM). Statistically significant differenced were analyzed by Kruskal–Wallis test. NK cells from peritoneum were also analyzed for the expression of the cytokines IFNγ, TNFα, the cytotoxicity-associated FasL and the proliferation marker KI-67 (**d**). At least six animals from two independent experiments were used for the analysis. Statistically significant differences were analyzed by an unpaired t test and were indicated as follows: *p < 0.05; **p < 0.01 and ***p < 0.001

To functionally characterize the possible memory-like NK cell population during FV infection, we challenged FV-infected mice with FV-derived tumor cells (FBL-3 cells expressing FV antigens) at 26 dpi (Fig. 2a). As control, we also injected FBL-3 cells into the peritoneum of naïve mice. After 48 h incubation, we re-isolated peritoneal cells and measured the killing of FBL-3 cells. Recipient mice were depleted for CD8^+^ T cells to prevent tumor cell killing by cytotoxic T cells. We detected a NK cell-mediated lysis of FBL-3 cells of around 25% in naïve mice, whereas we measured approximately 50% FBL-3 cell killing in FV-infected mice (Fig. 2b). NK cell-depleted mice were used in every experiment to calculate the killing mediated by NK cells. To address the question whether we can see an influx of NK cells into the peritoneum of naïve and previously FV-infected mice from the spleen and liver after FBL-3 challenge, we analyzed the absolute numbers of NK cells at 0, 6, 24 and 48 h after tumor cell injection (pooled data, Fig. 2c). NK cell numbers increased by 4-times at 6 h post FBL-3 cell injection, but subsequently decreased during the later time points (24 h, 48 h). Nevertheless, we detected significantly increased NK cell numbers at 48 h in comparison to the time point of challenge. We next analyzed whether these expanding NK cells showed an increased proliferation upon challenge with FBL-3 cells. We concentrated on the 48 h time point, because that was also the time point of the killing assay. Interestingly, the percentage of KI-67^+^ NK cells was not increased in FV-sensitized mice in comparison to naïve mice (Fig. 2d, Additional file 2: Fig. S2, KI-67). The KI-67 protein (also known as MKI67) is a cellular marker for proliferation. It is strictly associated with cell proliferation. Memory-like NK cells were shown to produce large quantities of the pro-inflammatory cytokines [7]. Thus, we asked whether peritoneal NK cells express cytotoxic molecules (FasL) and whether they produce Th1-associated cytokines (IFNγ, TNFα) (Fig. 2d, Additional file 2: Fig. S2). We detected a significantly higher percentage of IFNγ^+^ and TNFα^+^ NK cells in FV-infected compared to naïve mice challenged with FBL-3 cells. Although there were no differences in the percentage of FasL^+^ NK cells, we detected a significant increase of the mean fluorescence intensity (MFI) of FasL expression (Fig. 2d, Additional file 2: Fig. S2).

Here, we demonstrate the pro-inflammatory cytokine production and the cytotoxicity of memory-like NK cells during infection of mice with FV.

### NK cells from infected livers as well as from other FV-infected organs exhibit augmented antiviral activities in an adoptive transfer experiment

**Fig. 3:**
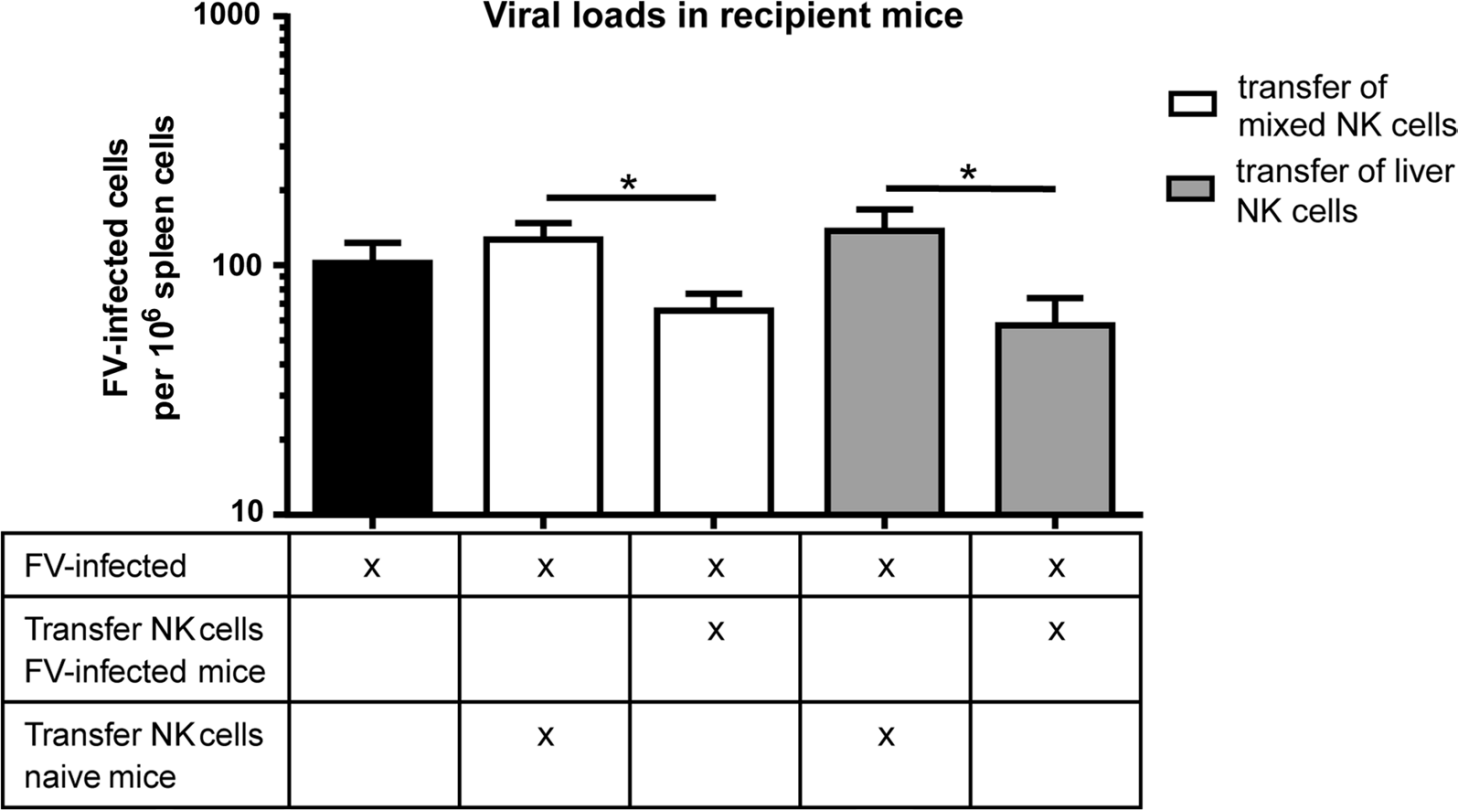
Effect of transferred memory-like NK cells on viral loads. Mice were naïve or infected with FV for 28 days. Bone marrow, spleens and lymph nodes were removed and cells were pooled. NK cells were isolated from pooled bone marrow, spleens and lymph nodes (mixed NK cells) or from the livers. Isolated NK cells were transferred i. v. into naïve C57BL/6 mice. Recipient mice were infected with FV at the same day. At 3 dpi, mice were sacrificed and viral loads were determined by an IC assay in the spleen. Mean values are shown, with SEM indicated by error bars. At least seven animals from at least three independent experiments were used for the analysis. Statistically significant differences were analyzed by an unpaired t test and were indicated by *p < 0.05

Memory-like NK cells appear to be resident in distinct organs, but the development and initiation of their adaptive features also depends on the initial pathogens [4, 6, 7, 17, 24]. Influenza infection as well as CHS result in the development of adaptive NK cells in the liver [4, 17, 24], whereas the existence of memory-like NK cells is also documented in spleens during CMV and SIV infection [7, 16]. To address the question whether NK cells from the liver only or also NK cells isolated from other lymphoid organs (pooled from spleen, lymph nodes and bone marrow) of FV-infected mice displayed memory-like functions, we analyzed viral loads after the transfer of NK cells into FV-infected recipient mice. FV replicates in the bone marrow, spleen and lymph nodes of infected mice [25], thus, NK cells from these organs had encountered virus-infected cells. We isolated NK cells from naïve or FV-infected mice and transferred cells into FV-infected recipient mice. In comparison to an FV-infected group without any cell transfer, we did not detect any differences in numbers of FV-infected cells after the transfer of naïve NK cells (Fig. 3). However, transfer of NK cells from FV-experienced mice resulted in a significant reduction in numbers of FV-infected cells in comparison to the transfer of NK cells from naïve mice. We also isolated NK cells only from liver tissues of FV-infected mice and naïve mice and transferred cells into FV-infected recipient mice. Interestingly, we also discovered significantly reduced numbers of FV-infected cells in the group receiving FV-sensitized NK cells in comparison to recipient mice that received naïve hepatic NK cells. Thus, we demonstrated that the adaptive functions of NK cells were not restricted to liver cells during FV infection, but could be found in hematopoietic organs too.

### FV-sensitized NK cells eliminate FV antigen-expressing target cells

**Fig. 4:**
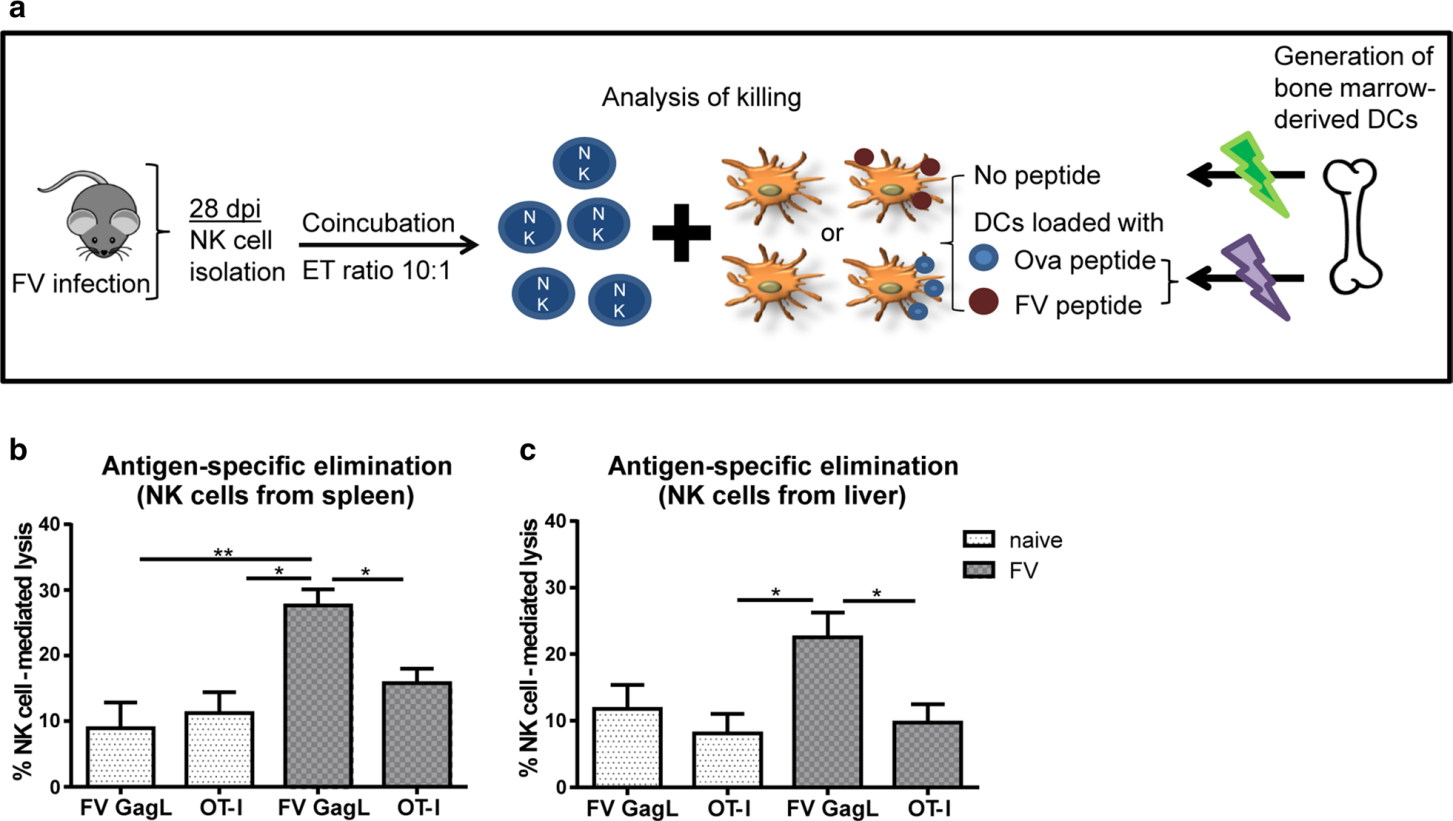
Antigen-specific elimination of peptide-loaded DCs by NK cells. Mice were infected with FV for 28 days. NK cells were isolated from spleens and livers of naïve (dotted white) or FV-infected mice (dotted grey). Bone marrow-derived DCs were generated from naïve C57BL/6 mice and were unloaded and loaded with FV GagL peptide or class I peptide epitope of ovalbumin (OT-I). Unloaded and either FV GagL peptide loaded or OT-I loaded DCs were mixed in a 1:1 ratio. NK cells were co-incubated with mixed DCs in an effector to target ratio of 10:1 (**a**). After 18 h, cells were measured at flow cytometer and specific lysis was calculated for splenic NK cells (**b**) and hepatic NK cells (**c**). A minimum of nine animals from at least four experiments were used for the analysis. Statistically significant differences were analyzed by a Kruskal–Wallis test (**b**) or an Ordinary one-way ANOVA (**c**) and are indicated by *p < 0.05 and **p < 0.01. Mean percentages are displayed by bars (± SEM)

According to classical textbooks NK cells recognize non-specifically MHC class I or MHC class I-like molecules on target cells with their germline-encoded receptors that lack an antigen-specific rearrangement, but recent studies have shown increasing evidences suggesting an antigen-specific recognition of target cells by NK cells [6, 11, 16]. In the MCMV infection model NK cells express the receptor Ly49H, which recognizes the virally encoded m157 protein on the surface of infected cells [26, 27]. Beside herpesvirus infection, Reeves and colleagues demonstrated an antigen-matched elimination of target cells in SIV or simian-human immunodeficiency virus (SHIV) infected macaques [16]. We addressed the question, if FV-sensitized NK cells preferentially eliminate target cells that were loaded with FV peptides on their MHC I molecules. To this end, we loaded bone marrow-derived DCs with the FV GagL peptide (the immunodominant CD8^+^ T cell epitope of FV), OT-I peptide or without a peptide and co-incubated them with NK cells isolated from 28 days FV-infected or naïve mice (Fig. 4a). We then analyzed and calculated the splenic (Fig. 4b) and hepatic (Fig. 4c) NK cell-mediated lysis of these DCs. We did not detect any substantial differences in the lysis (background at around 10%) of FV peptide or OT-I loaded DCs when co-incubated with NK cells from naïve mice. Interestingly, splenic NK cells from FV antigen-experienced mice eliminated up to 30% of FV peptide loaded DCs in comparison to only 16% of OT-I loaded DCs (Fig. 4b). Hepatic NK cells eliminated approximately 23% of the FV GagL peptide loaded DCs compared to the lysis of OT-I loaded DCs of around 10% (Fig. 4c).

These data demonstrate that NK cells from FV-infected mice eliminate target cells in an antigen-specific manner and thus develop a memory-like phenotype during infection.

Infections with viruses cause numerous health issues. Currently, 37 million people worldwide are living with the human immunodeficiency virus (HIV) that can cause a deadly immunodeficiency syndrome [28]. The antiretroviral therapy (ART) currently used for the treatment of HIV patients suppresses viral replication; however, a cure is still impossible. Therefore, further basic research is necessary to find a way to cure or functionally cure HIV infections and prevent AIDS-associated disease. Natural killer (NK) cells are one of the cytotoxic cell populations that are important for the control of acute retroviral infections [29, 30]. In this study, we analyzed the NK cell phenotype and functionality during the late phase of acute FV infection in the mouse. Our findings demonstrate the development and antigen-specific activity of memory-like NK cells during retroviral infection.

In the past years, memory-like NK cells have been studied using several mouse models [31, 32]. Memory-like NK cells can be phenotypically characterized analog to memory CD8^+^ T cells with the markers KLRG-1, CD27, CD62L, Ly-6c but also using CD11b, Thy-1 and CXCR6 [4, 6, 21, 23]. During FV infection, a higher percentage of NK cells expressed KLRG-1 and CXCR6, but not the other memory markers that have been described. CXCR6 is upregulated on hepatic NK cells, according to Paust and colleagues who described CXCR6 as a required chemokine receptor for memory-like liver NK cells in the CHS and virus-like particle (VLP) context [6].

The localization and the organ-associated function of adaptive NK cells can be different in different models. In models of CHS or influenza virus infection adaptive NK cells reside in the liver whereas an efficient memory response was also detectable within the spleen of MCMV-infected or SIV-infected animals. In FV infection, spleen-derived memory-like NK cells slightly better eliminated target cells upon second antigen encounter compared to hepatic NK cells. FV replicates efficiently in the spleen but very little virus is present in livers of FV-infected animals (data not shown). Thus, our findings indicate that the memory function of adaptive NK cells can develop independently of organ distribution but depend on the amount of viral antigens within these organs.

It was previously described that memory-like NK cells produce higher amounts of IFNγ and increase the expression of CD107a after secondary antigen encounter, indicating a direct cytotoxic function of NK cells after stimulation [7]. After the challenge of FV-experienced mice with FV-induced FBL-3 tumor cells (48 h), we also found an increased percentage of IFNγ and TNFα producing NK cells as well as a higher expression of FasL. We also detected an increased expression of granzyme B very early after challenge with FBL-3 cells (6 h, data not shown). These findings confirm the rapid secretion of pro-inflammatory cytokines as well as the direct cytotoxicity of memory-like NK cells towards secondary target cells.

NK cells recognize MHC I molecules with their inhibitory receptors, but it was also reported that the nature of the peptide, which is presented in the MHC binding groove, is of substantial importance for this recognition and may affect NK cell functions [13]. It was shown that amino acid changes in peptide sequences can abolish or trigger the binding of NK cells to target cells [33–35]. In the current study we show that FV antigen-experienced NK cells potently eliminate FV antigen-matched target cells. Beside studies in murine models, a study in a macaque SIV and SHIV infection model revealed that the recognition of target cells by memory NK cells is antigen-specific [16]. Moreover, NK cells from spleens and livers of macaques were both functional and able to lyse matched target cells similar to NK cells from FV model.

Taken together, we demonstrated the existence of memory-like NK cells during FV infection and their antigen-specific elimination of target cells upon secondary exposure to FV antigen. Overall, the FV mouse model is very powerful to study adaptive NK cells in order to get deeper insights into the molecular pathways of their induction and to find solutions for their clinical use. This might be important for cancer immunotherapy, the treatment of viral diseases or the improvement of vaccination strategies.

## Materials and methods

### Mice and infection

Inbred C57BL/6 mice were purchased from Harlan Envigo Laboratories and maintained under pathogen-free conditions. Experiments were performed using female C57BL/6 mice of the age of at least 7 weeks. Infection of mice was done with the FV complex containing B-tropic Friend murine leukemia helper virus and polycythemia-inducing spleen focus-forming virus. The FV stock was prepared as a 15% spleen cell homogenate from susceptible BALB/c mice infected 14 days previously with 3000 SFFU of FV. Mice were injected intravenously with 0.1 ml phosphate-buffered saline (PBS) containing 40,000 SFFU of FV, if not indicated differently. The virus stock was free of lactate dehydrogenase-elevating virus. Mice were sacrificed at 28 dpi by cervical dislocation.

### Flow cytometry

Single cell suspensions were prepared from spleens and livers. Erythrocytes were lysed in liver samples. After gradient centrifugation, hepatocytes were washed and used for stainings. Cells were stained with the following antibodies: CD3 (17A2), CD11b (M1/70), CD27 (LG.3A10), CD62L (MEL-14), CD69 (H1.2F3), CXCR-6 (SA051D1), FasL (MFL3), GzmB (GB11), IFNγ (XMG1.2), KI-67 (B56), KLRG-1 (2F1), Ly-6c (HK1.4), NK1.1 (PK136) or TNFα (MP6-XT22). Zombie UV Fixable Viability Kit (BioLegend) was used for the exclusion of dead cells. If stimulation was necessary, cells were stimulated with ionomycin (500 ng/ml), phorbol myristate acetate (PMA; 25 ng/ml), monensin (1X), and brefeldin A (2 µg/ml) diluted in Iscove’s modified Dulbecco’s medium (IMDM) buffer at 37 °C for 3 h. For intracellular stainings, cells were fixed with the Cytofix/Cytoperm Fixation/Permeabilization kit (BD) or with Foxp3/Transcription Factor staining buffer set (eBioscience). Cells were measured at LSR II (BD).

### Infectious center assay

Infectious centers (IC) were detected by tenfold serial dilution of single-cell suspensions onto *Mus dunni* cells. Co-cultures were incubated for 3 days and fixed with 96% ethanol. Fixed cells were washed twice with PBS plus bovine serum albumin (BSA) and stained with F-MuLV envelope-specific monoclonal antibody 720. After a second wash with PBS + BSA cells were incubated with peroxidase-conjugated goat anti-mouse antibody. For the detection of foci, assay was developed with aminoethylcarbazol.

### In vivo cytotoxicity assay

FV-derived tumor cells (FBL-3 cells) were fluorescently labeled and 5 × 10^5^ cells were injected i. p. into naïve or FV-infected mice at 26 dpi. Mice were injected i. p. with a CD8α-specific depletion antibody (YTS 169.4) 1 day prior FBL-3 injection and one day after application of FBL-3 cells. In control mice, also NK cell were depleted through i. p. injections of the NK1.1-specific monoclonal antibody PK136 one day prior FBL-3 injection and 1 day after injection. After 6, 24 or 48 h peritoneal cells were isolated through peritoneal lavage. Cells were stained and measured at LSR II. Target cell killing was calculated as previously described [36]:

$$\frac{{{\text{Target cells from NK cell depleted mice }} - {\text{Sample target cell number}}}}{\text{Target cells from NK cell depleted mice}} \times 100$$

### NK cell transfer

NK cells were isolated from spleen, bone marrow and lymph nodes (mix) or livers of mice according to the manufacturer’s protocol (MojoSort Mouse NK cell isolation kit, BioLegend). Purity of NK cell isolation was checked at LSR II (> 85%). The fraction of < 15% NK1.1-negative cells contained almost no B cells, T cells and granulocytes but DCs and macrophages. 5 × 10^5^ hepatic cells or 1 × 10^6^ mixed NK cells were transferred intravenously into naïve mice that were infected at the same day with 20,000 SFFU of FV. At 3 dpi, spleens were removed and viral loads were detected.

### In vitro antigen-specific NK cell killing assay

Based on the previously described protocol, the generation of bone marrow-derived DCs was done with some modifications [37]. In brief, cells were isolated from murine tibias and femurs and placed on petri dish plates containing 10 ml of DC media (RPMI supplemented with 10% FCS, 2 mM l-glutamine, 50 nM 2-mercapotoehanol, 1 mM sodium pyruvate, 0.5% penicillin/streptomycin, 5 ng/mL GM-CSF, and 1 ng/mL IL-4) and were incubated at 37 °C in a humidified 5% CO_2_ atmosphere. After 24 h, 10 ml of DC media was added. 15 ml of media was changed at day 3. At day 7, non-adherent cells were used for experiments. DCs were loaded either with FV GagL peptide (2 µg/ml), with the class I peptide epitope of ovalbumin (OT-I, 2 µg/ml) or without any peptide (unloaded) for at least 1 h at 37 °C. Unloaded DCs were stained with carboxyfluorescein succinimidyl ester (CFSE, 2.5 µM, BioLegend) whereas loaded cells were labeled with Tag-it Violet Proliferation and Cell Tracking Dye (2.5 µM, BioLegend). Loaded and unloaded cells were mixed in a 1:1 ratio. NK cells were isolated from spleens and livers according to the manufacturer’s instructions (MojoSort Mouse NK cell isolation kit, BioLegend). Purity of NK cell isolation was checked at LSR II (> 85%). Mixed DCs were co-cultured with NK cells in a 10:1 effector to target ratio for 18 h at 37 °C. Cells were immediately measured at LSR II. For calculation, the ratio of violet-labeled cells (loaded) versus CFSE-labeled cells (unloaded) was calculated and normalized to the ratio of mixed DCs without NKs.

### Statistical analyses

Statistical analyses were done with GraphPad Prism version 6. Statistical differences between two groups were analyzed by an unpaired t test (parametric) and differences between multiple groups were examined by Kruskal–Wallis test (non-parametric) or Ordinary one-way ANOVA (parametric).

## Additional files

**Additional file 1: Figure 1.** Expression of memory-associated NK cell markers after FV infection C57BL/6 mice were identified with FV and spleens and livers were collected at 28 dpi. Splenocytes and hepatocytes from naive mice were used as control. Representative dot plots are shown for the expression of CD11b, CD27, CD62L, Ly-6c, KLRG1 and CXCR6 by NK cells.

**Additional file 2: Figure 2.** Phenotype of NK cells after challenge with FBL-3 cells mice were naive or infected with FV for 26 days. FBL-3 cells were injected intraperitoneally and incubated for 2 days. Peritoneal lavage was performed and NK cells were stained for IFNγ, TNFα, FasL and KI-67 of peritoneal NK cells from naive and FV-infected mice.
