# Supplementary figures and images for: Friend retrovirus infection induces the development of memory-like natural killer cells
Source: Retrovirology. 2018 Oct 6;15:68. doi: 10.1186/s12977-018-0450-1 (PMC6174066; doi:10.1186/s12977-018-0450-1)

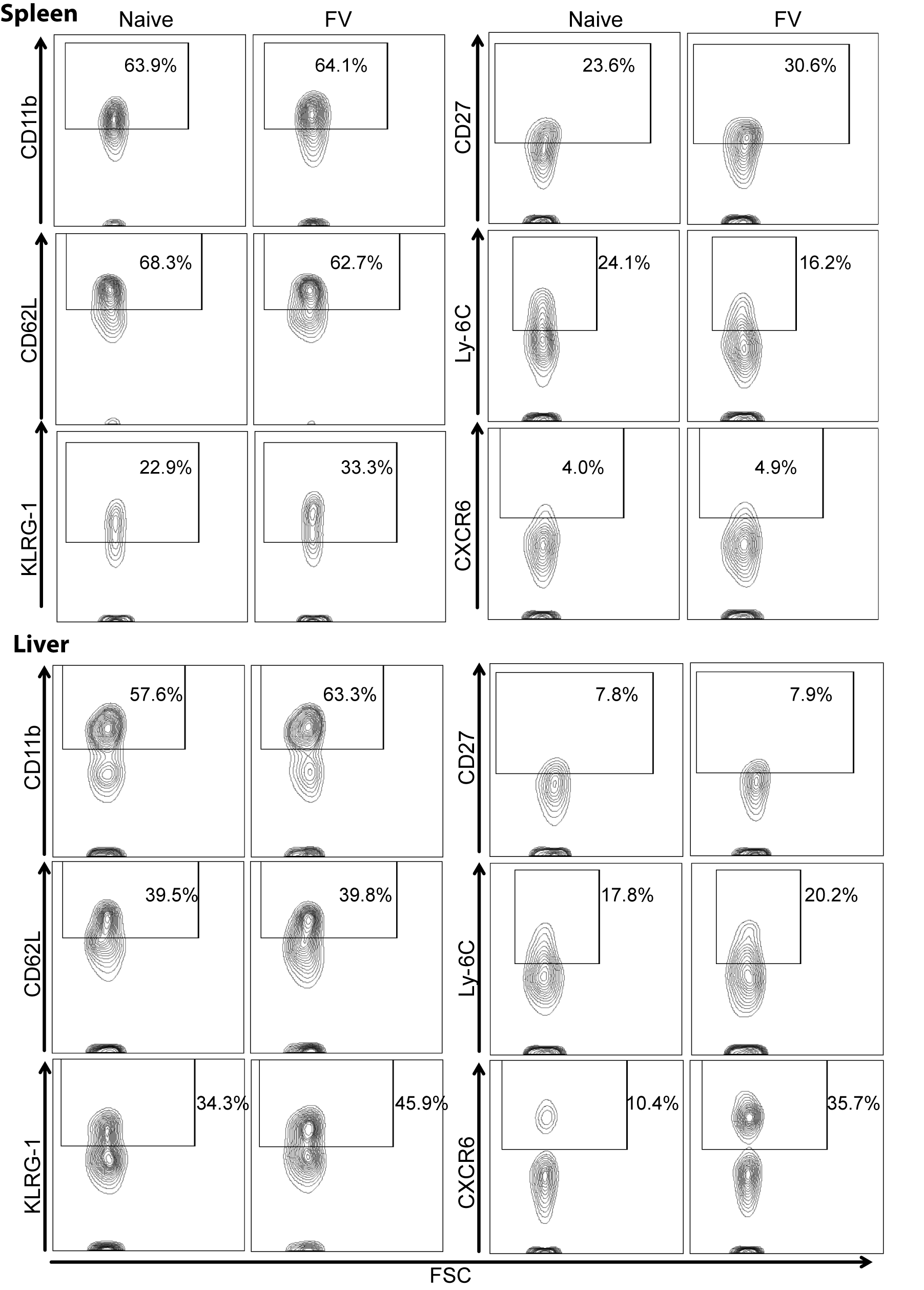

Supplement: Supplementary file 1 — Additional file 1: Figure 1. Expression of memory-associated NK cell markers after FV infection C57BL/6 mice were identified with FV and spleens and livers were collected at 28 dpi. Splenocytes and hepatocytes from naive mice were used as control. Representative dot plots are shown for the expression of CD11b, CD27, CD62L, Ly-6c, KLRG1 and CXCR6 by NK cells. [file 12977_2018_450_MOESM1_ESM.tif]

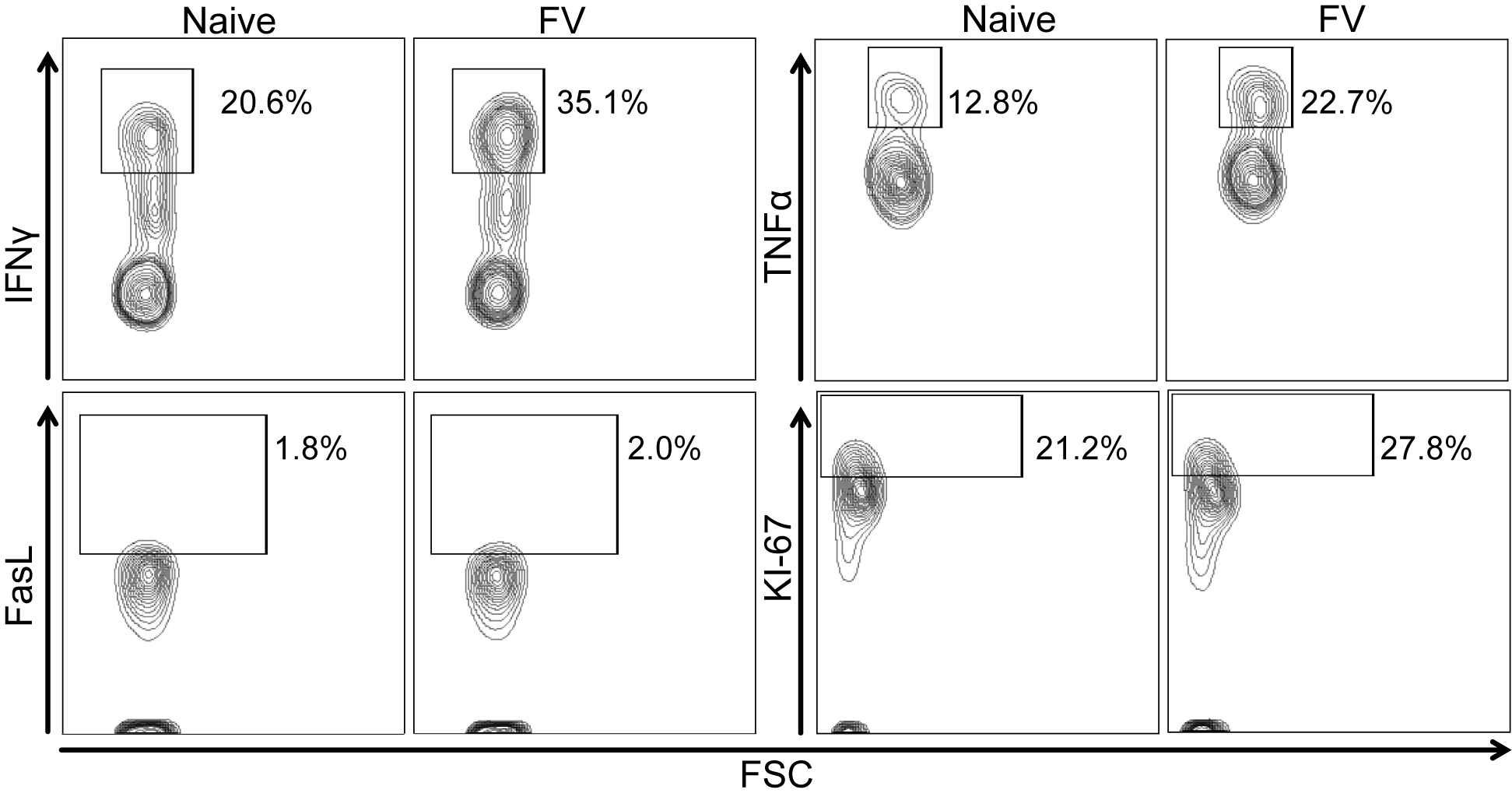

Supplement: Supplementary file 2 — Additional file 2: Figure 2. Phenotype of NK cells after challenge with FBL-3 cells mice were naive or infected with FV for 26 days. FBL-3 cells were injected intraperitoneally and incubated for 2 days. Peritoneal lavage was performed and NK cells were stained for IFNγ, TNFα, FasL and KI-67 of peritoneal NK cells from naive and FV-infected mice. [file 12977_2018_450_MOESM2_ESM.tif]
